# Exploration of the selective binding mechanism of protein kinase Aurora A selectivity via a comprehensive molecular modeling study

**DOI:** 10.7717/peerj.7832

**Published:** 2019-10-22

**Authors:** Zhe Zhang, Yafei Xu, Jian Wu, Ying Shen, Hao Cheng, Yiming Xiang

**Affiliations:** 1 Department of Surgery, Clinical Medical College, Hubei University of Science and Technology, Xianning, Hubei, China; 2 Department of Orthopedics, Nanhai Hospital, Southern Medical University, Foshan, Guangdong, China; 3 Department of Orthopedics, Xianning Central Hospital, Xianning, Hubei, China; 4 Department of Public Health, Xianning Central Hospital, Xianning, Hubei, China; 5 Department of Surgery, Second Affiliated Hospital of Hubei University of Science and Technology, Xianning, Hubei, China

**Keywords:** Aurora A, Enhanced simulation, Selective mechanism, Gaussian accelerated Molecular dynamics simulations, Conventional Molecular dynamics simulation, Umbrella sampling simulation

## Abstract

**Background:**

The kinase of Aurora A has been regarded as a promising therapeutic target due to its altered expression in various human cancers. However, given the high similarity of the active binding site of Aurora A to other kinases, designing highly selective inhibitors towards Aurora A remains a challenge. Recently, two potential small-molecule inhibitors named AT9283 and Danusertib were reported to exhibit significant selectivity to Aurora A, but not to Gleevec. It was argued that protein dynamics is crucial for drug selectivity to Aurora A. However, little computational research has been conducted to shed light on the underlying mechanisms.

**Methods:**

In this study, MM/GBSA calculations based on conventional molecular dynamics (cMD) simulations and enhanced sampling simulations including Gaussian accelerated MD (GaMD) simulations and umbrella sampling were carried out to illustrate the selectivity of inhibitors to Aurora A.

**Results:**

The calculation results from cMD simulation showed that the binding specificity is primarily controlled by conformational change of the kinase hinge. The protein dynamics and energetic differences were further supported by the GaMD simulations. Umbrella sampling further proved that AT9283 and Danusertib have similar potential of mean force (PMF) profiles toward Aurora A in terms of PMF depth. Compared with AT9283 and Danusertib, Gleevec has much lower PMF depth, indicating that Gleevec is more easily dissociated from Aurora A than AT9283 and Danusertib. These results not only show the selective determinants of Aurora A, but also provide valuable clues for the further development of novel potent Aurora A selective inhibitors.

## Introduction

Aurora A, a member of the serine/threonine kinase family, is a key mitotic regulator that plays an essential role in the maintenance of chromosomal stability. It has been proposed that Aurora A is dysregulated in numerous cancer cells, including breast cancer, metastatic colorectal cancers, non-small-cell lung cancer (NSCLC), osteosarcoma, among others (Damodaran et al., 2017; Yan et al., 2016). In addition, Aurora A helps to develop resistance to standard chemotherapy by providing cells with stem-like properties. More specifically, the transformation Aurora A cells from epithelial to mesenchymal type will promote the progression of tumor metastasis, and increase resistance to standard treatments (Cammareri et al., 2010; Zheng et al., 2014). These vital properties make Aurora A a highly promising potential target for developing small-molecule inhibitors against various cancers. Already, many of these inhibitors have entered early-phase clinical trials.

**Figure 1:**
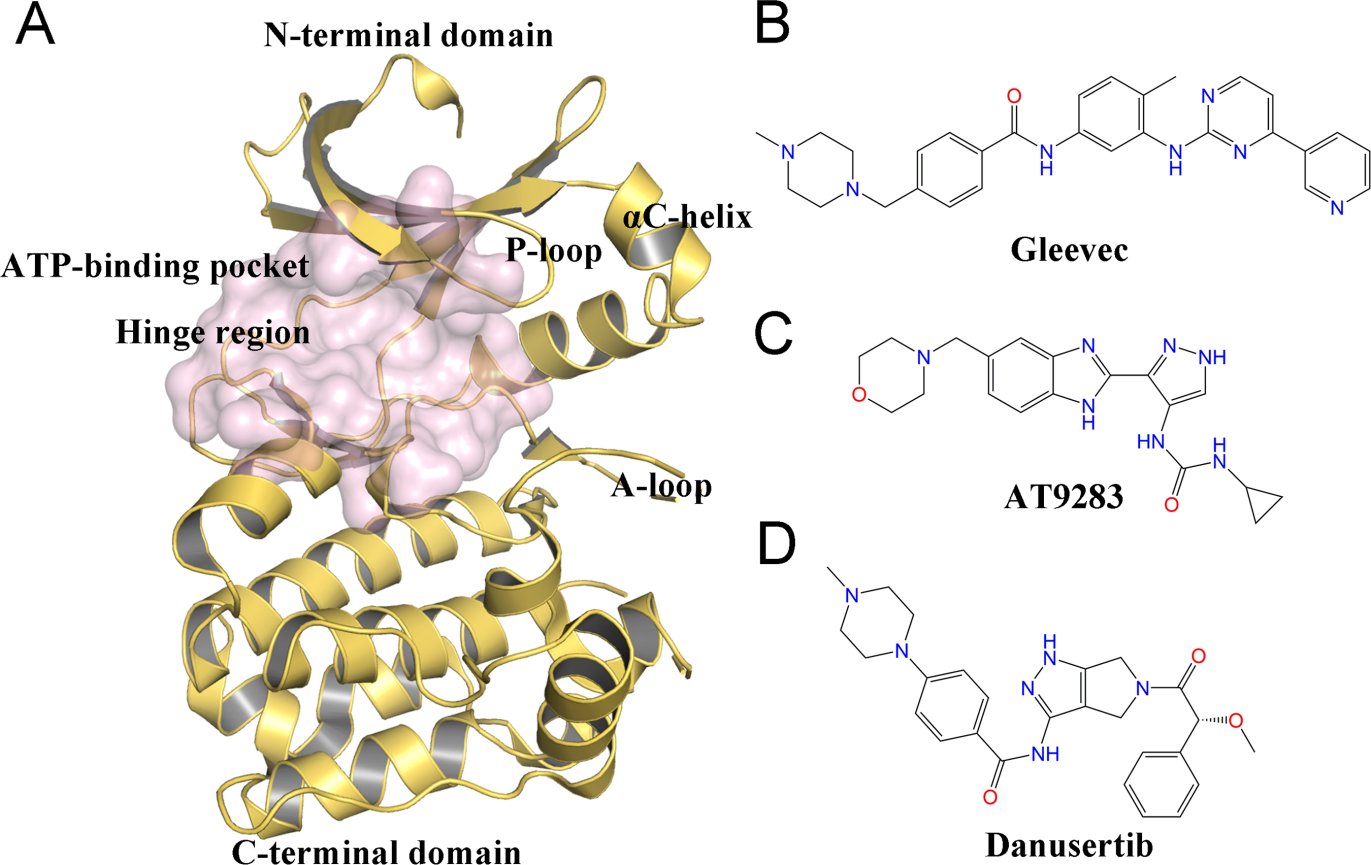
Overview of the structure of Aurora A and chemical structures of three small-molecules in this work. (A) Overview of the Aurora A structure, the active binding pocket is colored light magenta. The chemical structures of Gleevec (B), AT9283 (C) and Danusertib (D).

Despite the therapeutic value of Aurora A inhibitors, the development of specific kinase inhibitors has proved to be challenging. This is largely due to the fact that they must discriminate among a large number of other kinases, which have highly similar active sites in human cells. Similar to other kinases, Aurora A consists of a short N-terminal domain and a highly evolutionary conserved C-terminal catalytic domain (Fig. 1A). The N-terminal domain consists of a five-stranded antiparallel *β* sheet, an important regulatory *α*C-helix, and a glycine-rich loop (P-loop). The C-terminal domain is mostly *α* helical and contains the activation loop (A-loop) involved in polypeptide substrate binding. These two domains are connected by a flexible joint called kinase hinge, with the nucleotide binding pocket located in between. Up to now, a large number of kinase inhibitors have been developed. However, only a few of them can specifically target Aurora A. Thus, understanding the selective mechanisms of the protein-ligand recognition at the molecular level may provide valuable information for rational drug design of selective Aurora A inhibitors and help to mitigate potential side effects.

Molecular dynamics (MD) simulation analysis has proved to be a powerful and valuable tool for obtaining comprehensive information about various kinases, such as Anaplastic lymphoma kinase (ALK), breakpoint cluster region and the Ableson (BCR-Abl), and epidermal growth factor receptor (EGFR) (Bello, 2018; Kong et al., 2018; Zhang et al., 2019). Although some dynamic behaviors between Aurora A and inhibitors have been investigated by MD simulations, few studies have sought to elucidate the detailed selective mechanisms (Cheng et al., 2011; Oliveira, Ahmad & Engh, 2011; Talele & McLaughlin, 2008; Yang et al., 2012). In this study, three ATP-competitive inhibitors named AT9283, Danusertib, and Gleevec were used to study the drug selective mechanisms of Aurora A (Figs. 1B–1D). AT9283 is a multi-targeted kinase inhibitor of Aurora kinases (Aurora A and B) as well as other kinases, including BCR-Abl, Fms-like tyrosine kinase (FLT3), and Janus Kinase 2 (JAK2) (Howard et al., 2009). AT9283 has also entered several clinical trials and demonstrated significant Aurora kinase inhibition at tolerable doses with disease stabilization in adults and children with solid tumors (Borisa & Bhatt, 2017). Danusertib (formerly known as PHA-739358) is a pan-Aurora kinase (Aurora A, B and C) inhibitor, and was one of the first Aurora kinase inhibitors to enter phase I and II clinical trials for the treatment of Leukemia, Multiple Myeloma (Falchook, Bastida & Kurzrock, 2015). Gleevec (known as imatinib) has a significant therapeutic effect in the early stages of chronic myeloid leukemia (CML), targeting the BCR-Abl tyrosine kinase (TK) (Kantarjian et al., 2012). Recently, using a combination of biophysical techniques, Pitsawong et al. (2018) studied how three well-known anti-cancer drugs interact with Aurora A. Two of them (AT9283 and Danusertib) specifically switched off Aurora A, but not Gleevec. They proposed that AT9283 and Danusertib specifically target Aurora A, which works by inducing fit effects rather than conformational selection. In contrast, no induced fit effect was observed for Gleevec on Aurora A, which thus binds less tightly to Aurora A. However, more research is needed to characterize the dynamic behavior and free-energy map (FEM) to elucidate the detailed selective mechanisms of drugs for Aurora A (Pitsawong et al., 2018).

Herein, conventional molecular dynamics (cMD) simulations along with structural analysis and two-end-state free energy calculations were carried out to elucidate the selective mechanisms underlying the conformational and energetic differences between each drug. Then, Gaussian accelerated molecular dynamics (GaMD) simulations in conjunction with structural analysis, principal component analysis (PCA) and 2D free energy calculations were performed to sample more conformational space, because cMD simulation remains a set of limited conformational ensembles. Furthermore, umbrella sampling (US) simulations were performed to investigate the dissociation pathways of drugs from the binding pockets of Aurora A. These comprehensive molecular modeling results may provide valuable guidance for the selectivity optimization of candidate drugs.

## Materials & Methods

### Preparation of the studied systems

The crystal structures of human Aurora A/AT9283 (PDB code: 6CPG) and Aurora A/Danusertib (PDB code: 2J50) were obtained from The *Protein Data Bank* (PDB) web server (Smith et al., 2015). The missing side-chains and loop structures in the crystal structures of Aurora A were refined using the *Loops/Refine Structure* module in UCSF Chimera program (Pettersen et al., 2004). The protonation states of the Aurora A residues were estimated using PDB2PQR web Server (Dolinsky et al., 2004). The charge state of the Gleevec, AT9283 and Danusertib were set as +1, 0, 0, respectively. The initial structure of Aurora A/Gleevec was implemented using molecular docking in the AutoDock 4.2 program (Morris et al., 2009). A cubic box of 25 Å × 25 Å × 25 Å was defined, with ATP-binding sites as the center and a grid spacing of 0.3 Å. The AutoDockTools program was used to distribute Gasteiger partial charges to the atoms of Gleevec (Morris et al., 2009). AutoGrid software was applied to estimate the affinity maps of Aurora A. The docking parameters were set as follows: trials of 300 dockings and clustered according to the RMSD tolerance of 1.0 Å, maximum number of evaluations was set as 25,000,000, and other parameters were set at default values. The highest ranking structure for Aurora A/Gleevec was selected for further molecular dynamics (MD) simulation protocols.

### Conventional MD simulation

Conventional MD (cMD) simulations were performed using Assisted Model Building with Energy Refinement (Amber) 16 software. The restrained electrostatic potential (RESP) method was utilized to calculate the partial atomic charges for AT9283, Danusertib and Gleevec based on HF/6-13G* basis set. Then, the Amber ff14SB force field and the General Amber Force Field (GAFF) in the LEaP modules were implemented to describe the Aurora A and ligands in Amber 16 software, respectively (Maier et al., 2015; Wang et al., 2004). Subsequently, these systems were solvated in a cubic box of TIP3P water molecules with the distance between the complex surface and the box boundary set at 20 Å. To ensure the overall charge neutrality, an appropriate number of counter ions were added.

A three-step minimization protocol was implemented to optimize the systems prior to the cMD simulations. Firstly, the 6,000-step steepest descent (SD) method was utilized to minimize the water molecules. This was followed by the implementation of the 6,000-step conjugate gradient (CG) method in order to keep the proteins in a position other than the hydrogens. Then, the same minimization scheme was applied to optimize the protein side chains. At last, the whole system was relaxed for the 10,000-step SD method followed by the 6,000-steps CG method without any restraints. Then, the optimized systems were progressively heated from 0 K to 100 K and then to 310 K with the protein restrained over 300 ps in the canonical (NVT) ensemble. To accommodate solvent density, the whole system was equilibrated over 500 ps at a constant pressure and for 5 ns in the isothermal isobaric (NPT) ensemble. Finally, the unrestrained 500 ns cMD simulations were carried out in the NPT ensemble to produce trajectories with the temperature set at 310 K. During the simulation process, temperature was regulated by Langevin dynamics and the pressure was controlled using *Berendsen barostat* (Izaguirre et al., 2001; Larini, Mannella & Leporini, 2007). *Particle-mesh Ewald* (PME) was used to consider the long-range electrostatics with a cutoff value of 10 Å for the non-bonding interactions, and the *SHAKE* method was selected to maintain the rigidity of all bonds (Essmann et al., 1995; Krautler, Van Gunsteren & Hunenberger, 2001). The time step was set to 2 fs, and the trajectory frames were saved at an interval of 10 ps for further analysis.

### Dynamic cross-correlation (DCC) analysis

To better understand the dynamic behavior of the simulated systems, DCC analysis was conducted to evaluate the cross-correlation displacement of the protein backbone atoms (C_α_). The cross-correlation matrix (*C*_*ij*_) between residues *i* and *j* were calculated using the Bio3D package in R (Skjaerven et al., 2014). The *C*_*ij*_ was determined by the following equation:

(1) \begin{eqnarray*}{C}_{ij}= \frac{ \left\langle \Delta {r}_{i\cdot }\Delta {r}_{j} \right\rangle }{\sqrt{ \left\langle \Delta {r}_{i}^{2}\Delta {r}_{j}^{2} \right\rangle }} \end{eqnarray*}

where Δ*r*_*i*_ or Δ*r*_*j*_ represents the displacement from the mean position of the *i*th or *j*th atom, and angle bracket denotes an average over the sampled period. In this study, only *C*_*α*_ was applied for analysis by averaging motions of *C*_*α*_ atoms deviating from the mean structure based on last 200 ns trajectories from cMD and GaMD simulations with a total of 5,000 snapshots for each method.

### Principal component analysis (PCA)

PCA was performed to study the overall motion of Aurora A protein in each simulated system. It was performed through a commonly used protocol, which involved the elimination of the translational and rotational motions of the protein C_α_ atoms by aligning the structures from cMD/GaMD simulation trajectories. Then, the 3N ×3N covariance matrix was created using Cartesian coordinates, followed by construction of eigenvectors by diagonalization of the covariance matrix. Herein, PCA was calculated using the trajectories from the 500 ns cMD simulation and 600 ns GaMD simulation trajectories.

### Thermodynamic calculations

In accordance with a standard protocol reported in previous studies, the binding free energies and per-residue contribution to ligands were calculated using the Molecular Mechanics/Generalized Born Surface Area (MM/GBSA) method based on the single cMD simulation trajectory (Piao et al., 2019; Sun et al., 2018; Zheng et al., 2018). The Δ*G*_binding_ is given by:

(2) \begin{eqnarray*}& & {G}_{binding}={G}_{R+L}-({G}_{R}+{G}_{L})\end{eqnarray*}

(3) \begin{eqnarray*}& & \left\langle G \right\rangle = \left\langle {E}_{\mathrm{MM}} \right\rangle + \left\langle {G}_{\mathrm{sol}} \right\rangle -T \left\langle {S}_{\mathrm{MM}} \right\rangle \end{eqnarray*}

(4) \begin{eqnarray*}& & {E}_{\mathrm{MM}}={E}_{\mathrm{int}}+{E}_{\mathrm{vdW}}+{E}_{\text{elec}}\end{eqnarray*}

(5) \begin{eqnarray*}& & {G}_{\mathrm{sol}}={G}_{\mathrm{GB}}+{G}_{\mathrm{SA}}\end{eqnarray*}

where Δ*G*_*R*+*L*_, Δ*G*_*R*_, and Δ*G*_*L*_ represent the free energies of the receptor–ligand complex, receptor, and ligand, respectively (Eq. 2). The free energy term is calculated as an average over the ensemble of time-equidistant snapshots (Eq. 3). The molecular mechanical energy (*E*_MM_) can be divided into three terms (Eq. 4): intermolecular energy (*E*_int_), van der Waals energy (*E*_vdW_), and electrostatic energy (Δ*E*_elec_). The solvation term (*G*_solv_) consists of polar (*G*_SA_) and nonpolar contributions (*G*_GB_). In this study, the *G*_GB_ was estimated using the Generalized Born (GB) model reported by Onufriev, Bashford & Case (2004) (igb = 2); the dielectric of solute and solvent were set to 1 and 80, respectively. The *G*_SA_ is estimated using a fast linear combination of the pairwise overlap (LCPO) model with a probe radius of 1.4 Å. Conformational entropy (−TΔ*S*) was not considered due to the high computational demand and low accuracy (Hou et al., 2011). A total of 2,000 snapshots provided by last 200 ns simulation trajectories were used for binding free energy and per-residue energy contribution calculations.

### Gaussian accelerated MD (GaMD) simulations

The equilibrated snapshots extracted from cMD simulations served as the initial structures for the GaMD simulations. In our study, dual-boost GaMD simulations were carried out, which means that both the dihedral energy and total potential energy of the system were added to the simulated systems. The threshold energy was set to the lower bound (*E* = *V*_max_), and the average and standard deviation (Std) of the simulated system potential energies were calculated every 200 ps. The upper limits of the boost potential Std of both the dihedral and total potential energies were set as 6.0 kcal/mol. Herein, a 10 ns cMD simulation was used to collect the maximum, minimum, average, and Std values of the potential of simulated system for obtaining the acceleration parameters of GaMD. Finally, 600 ns GaMD simulations were carried out with random atomic velocity initializations. During the simulation, temperature was regulated by Langevin dynamics and pressure was controlled using *Berendsen barostat* (Izaguirre et al., 2001; Larini, Mannella & Leporini, 2007). *Particle-mesh Ewald* (PME) was used to consider the long-range electrostatics with a cutoff of 10 Å for the non-bonded interactions, and the *SHAKE* method was adopted to maintain all bonds rigid (Essmann et al., 1995; Krautler, Van Gunsteren & Hunenberger, 2001). The time step was set to 2 fs, and the trajectory frames were saved at an interval of 20 ps for further analysis. After the GaMD simulation, the principal component 1 (PC1) and principal component 2 (PC2) calculated from PCA in conjunction with the potential boost provided by GaMD simulations were performed to obtain the original free energy map (FEM) by the cumulant expansion to the second order method (Miao et al., 2014; Roe & Cheatham 3rd, 2013).

### Umbrella sampling (US) simulations

The equilibrated structures extracted from the trajectories from MD simulations served as the initial structures for the umbrella sampling simulations. The direction of the reaction coordinates (RCs) along the largest binding pocket was determined using the CAVER Analyst 1.0 software (Kozlikova et al., 2014). Subsequently, the distance between one atom in Aurora A (*C*_*α*_ of His-187) and another carbon atom in ligand was selected as the RCs (Fig. S1). In this study, the RCs of the studied systems were extended 20 Å from the initial position unbound from ATP binding pocket. For each system, the RCs were separated into 41 windows, each with a length of 0.5 Å. The initial conformation for each window was from the last snapshot of the previous window and each window was simulated with an 8 ns umbrella sampling simulation to ensure system convergence. A constant force of 10 kcal mol^−1^ Å^−2^ was added to each ligand to pull away from the binding pocket. The weighted histogram analysis method (WHAM) was used to calculate the potential of mean force (PMF), and the biased probability distribution was converted into a normal distribution (Kumar et al., 1992).

**Figure 2:**
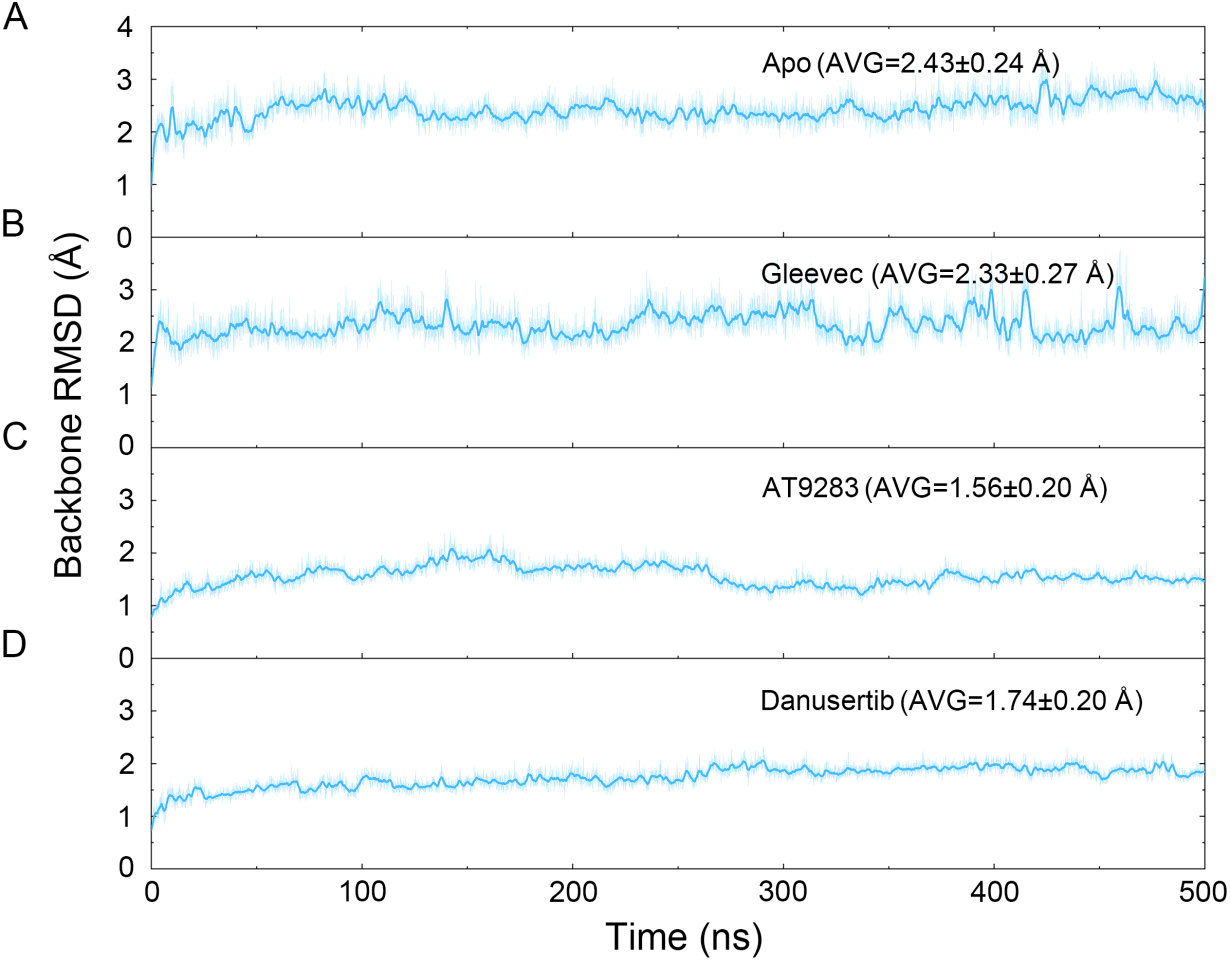
Time evolution of the RMSD values of backbone atoms of the Aurora A protein with (Gleevec, AT9283 and Danusertib) or without (apo) ligand in the simulated systems from cMD simulations. (A) Apo Aurora A; (B) Aurora A/Gleevec; (C) Aurora A/AT9283; (D) Aurora A/Danusertib.

## Results and Discussion

### Structural equilibrium and flexibility of the studied systems

**Figure 3:**
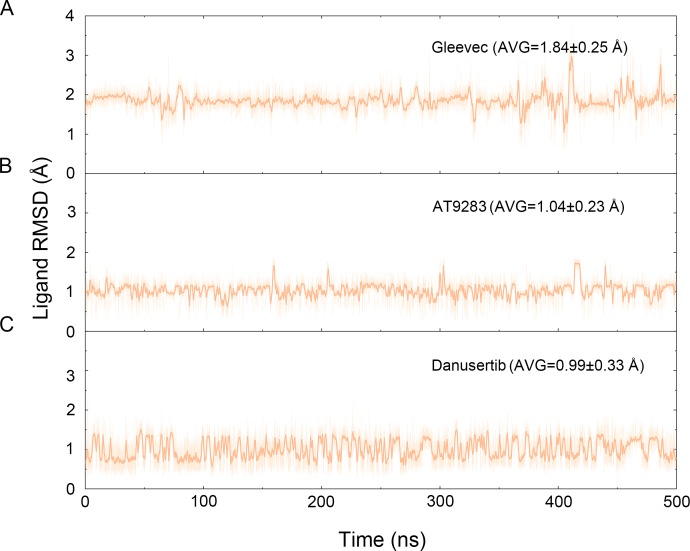
Time evolution of the RMSD values of heavy atoms of Gleevec, AT9283 and Danusertib bound with Aurora A in the simulated systems from cMD simulations. (A) Aurora A/Gleevec; (B) Aurora A/AT9283; (C) Aurora A/Danusertib.

The binding mode from molecular docking prompted us to explore the dynamic behavior of the simulated complexes. To analyze the stability and flexibility of the system, the root-mean square deviations (RMSDs) of the C_α_ atoms relative to their original position were monitored. As plotted in Fig. 2, the RMSD evolution from the cMD simulations of the backbone atoms (*C*_*α*_) of the Apo Aurora A, Aurora A/Gleevec, Aurora A/AT9283, Aurora A/Danusertib protein tended to converge after ∼50–300 ns, indicating that the simulated systems achieved sufficient stability through 500 ns of cMD simulations. Accordingly, the RMSD evolution of the heavy atoms of the Gleevec, AT9283, Danusertib in each simulated system maintained relative stability (RMSD fluctuation <2 Å) during the 500 ns simulation (Fig. 3). Interestingly, the average RMSD values of Aurora A bound with/without different ligands from cMD simulations follow the order of Apo >Gleevec >Danusertib >AT9283. Similarly, ligands that follow the order of Gleevec >AT9283 >Danusertib showed similar values. These results were in direct agreement with the experimental data that selective ligands of AT9283 and Danusertib were more stable in Aurora A than the non- selective ligand of Gleevec. Moreover, the apo Aurora A exhibited more fluctuation during the whole cMD simulation, suggesting that apo Aurora A or Aurora A bound with non-selective ligand may undergo dramatic conformational fluctuations that cannot stabilize the protein conformation. Therefore, the structural and energetic properties of simulated systems were analyzed during the last 200 ns cMD simulation trajectories.

**Figure 4:**
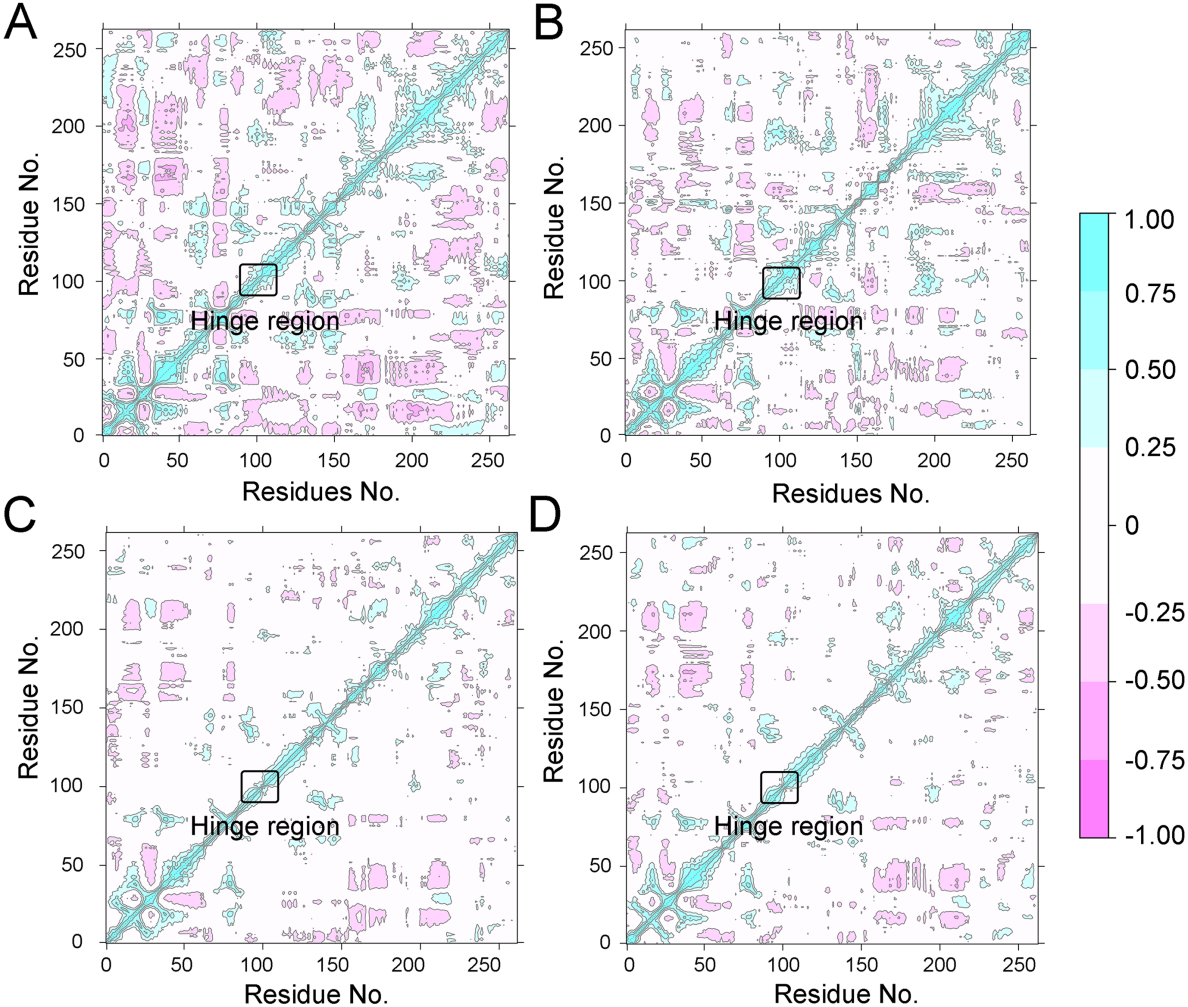
Dynamical cross-correlation (DCC) analysis of fluctuations of residues from cMD simulations. (A) Apo Aurora A; (B) Aurora A/Gleevec; (C) Aurora A/AT9283; (D) Aurora A/Danusertib.

To further investigate the effects of selective and non-selective inhibitors on the structural movements of Aurora A, principal component analysis (PCA) and dynamic cross-correlation (DCC) analysis were carried out. As plotted in Fig. 4, the correlation ranges from −1 to 1, corresponding to the extent of correlation or inverse correlation as indicated by the respective color intensities. Blue color represented positive correlation values ranging from 0.25 to 1; magenta color represented inverse correlation values ranging from −0.25 to −1; and light blue or magenta color represented weak or no-correlation for values ranging from −0.25 to +0.25. As shown in Figs. 4A–4B, the blue and magenta regions for Apo Aurora A and Aurora A/Gleevec were quite similar. A similar pattern was observed for Aurora A bound with AT9283 or Danusertib (Figs. 4C–4D). However, those regions in the Apo Aurora A and Aurora A/Gleevec were larger and more intense than those of Aurora A/AT9283 and Aurora A/Danusertib. These results present evidence that the overall correlated motions of Apo Aurora A and Aurora A/Gleevec are more dramatic than those of Aurora A/AT9283 and Aurora A/Danusertib, suggesting that the correlation or inverse correlation motions were enhanced in the Apo Aurora A and Aurora A/Gleevec. Besides, binding of Apo Aurora A or Aurora A with non-selective inhibitor may be more pronounced than binding with selective inhibitor.

**Figure 5:**
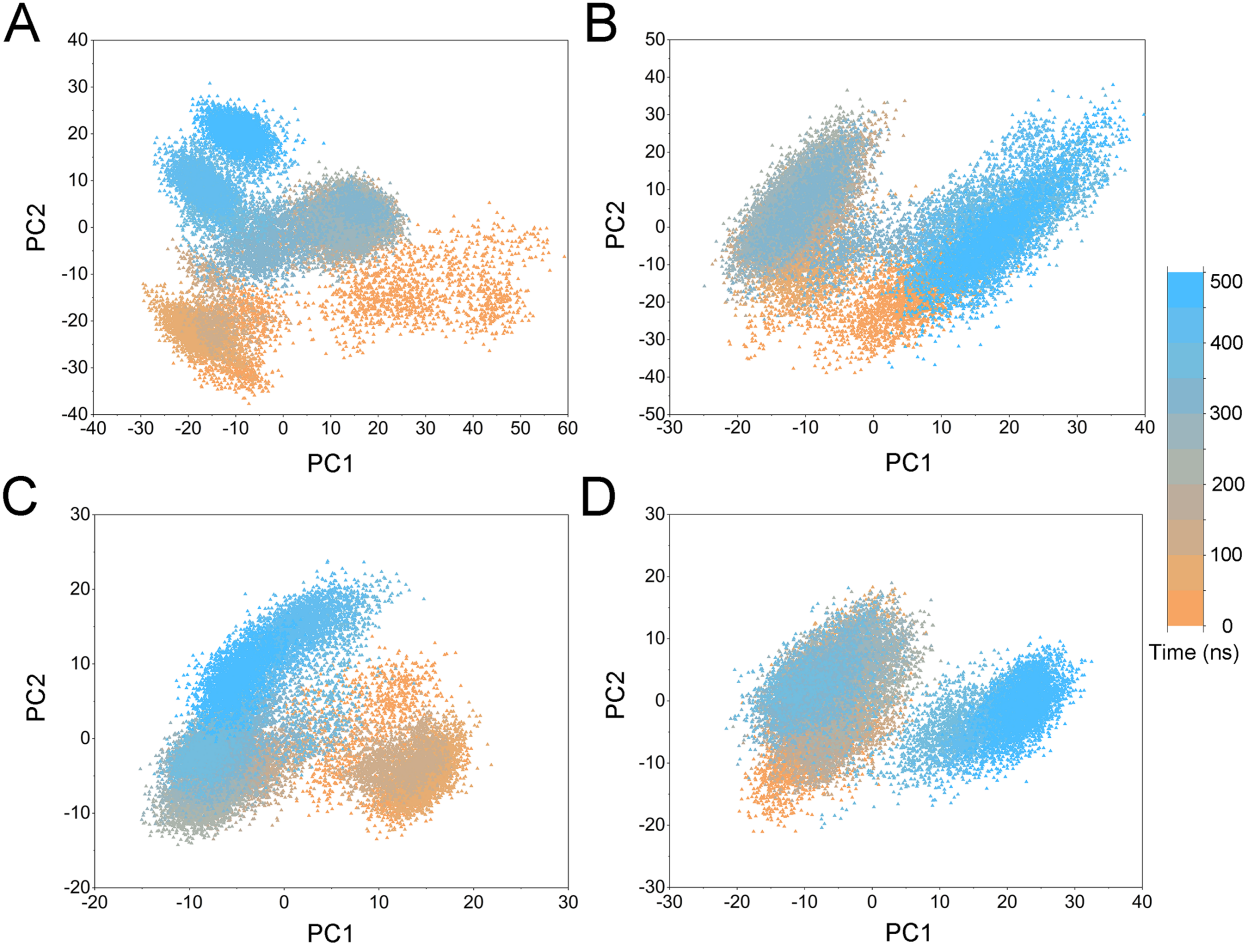
PCA scatter plot along the first two principal components from cMD simulations. (A) Apo Aurora A; (B) Aurora A/Gleevec; (C) Aurora A/AT9283; (D) Aurora A/Danusertib.

To highlight the differences of Aurora A’s collective motion in different systems, PCA was performed to further characterize the conformational transformations over time. PCA was commonly utilized to reduce the dimensionality of the MD simulation data and to identify conformational transformations. In theory, the first two principal components (PC1 and PC2) from PCA capture the majority of the variance in the original distribution of molecule conformational ensembles. Herein, the conformational ensembles of the systems under study were analyzed by projecting the trajectories of PC1 and PC2 into a two-dimensional space. When PC1 and PC2 are mapped onto each other, structures with a high degree of similarity cluster together. Therefore, each cluster represents a different protein conformational state. As illustrated in Fig. 5, the simulated conformations in each system were dynamic and fluctuant during 500 ns cMD simulations, and eventually stabilizes into a dominant state. The conformational changes of the Apo Aurora A and Aurora A/Gleevec were greater than those of the Aurora A/AT9283 and Aurora A/Danusertib. This demonstrated that the conformational distributions of Aurora A bound without/with non-selective inhibitor (Apo Aurora A and Aurora A/Gleevec) were remarkably different from those bound with selective inhibitors. Then, the frequencies of PCA scatter plots were quantified and the highest-frequency structures were aligned to their initial structures for the four simulated systems as plotted in the Figs. S2 and S3. Surprisingly, these representative structures did not undergo large conformational changes (except the flexible loops, such as A-loop, Fig. S3). These results indicated that Aurora A bound with selective inhibitors had a different conformational fluctuation compared to that bound with non-selective. Apo Aurora A or Aurora A bound with non-selective ligand may undergo dramatic conformational fluctuation that cannot stabilize the protein conformation. In comparison, Aurora A bound with selective ligand could stabilize its conformation, which is congruent with the DCC analysis results.

**Table 1:** Energy terms of Gleevec, AT9283 and Danusertib towards Aurora A (kcal/mol).

Name	Δ*E*_vdW_^a^	Δ*E*_elec_^b^	Δ*G*_GB_^c^	Δ*G*_SA_^d^	Δ*G*_binding_^e^
Aurora A/Gleevec	−40.16 ± 3.44	−25.34 ± 4.72	50.33 ± 3.21	−6.22 ± 0.35	−21.39 ± 3.11
Aurora A/AT9283	−45.97 ± 3.22	−35.98 ± 5.86	46.76 ± 3.77	−5.47 ± 0.28	−40.47 ± 2.90
Aurora A/Danusertib	−38.31 ± 2.99	−36.71 ± 4.61	41.94 ± 3.55	−5.91 ± 0.31	−38.99 ± 3.82

**Notes.**

a Van der Waals energy.

b Electrostatic energy.

c Electrostatic contribution to solvation.

d Non-polar contribution to solvation.

e Binding free energy.

**Figure 6:**
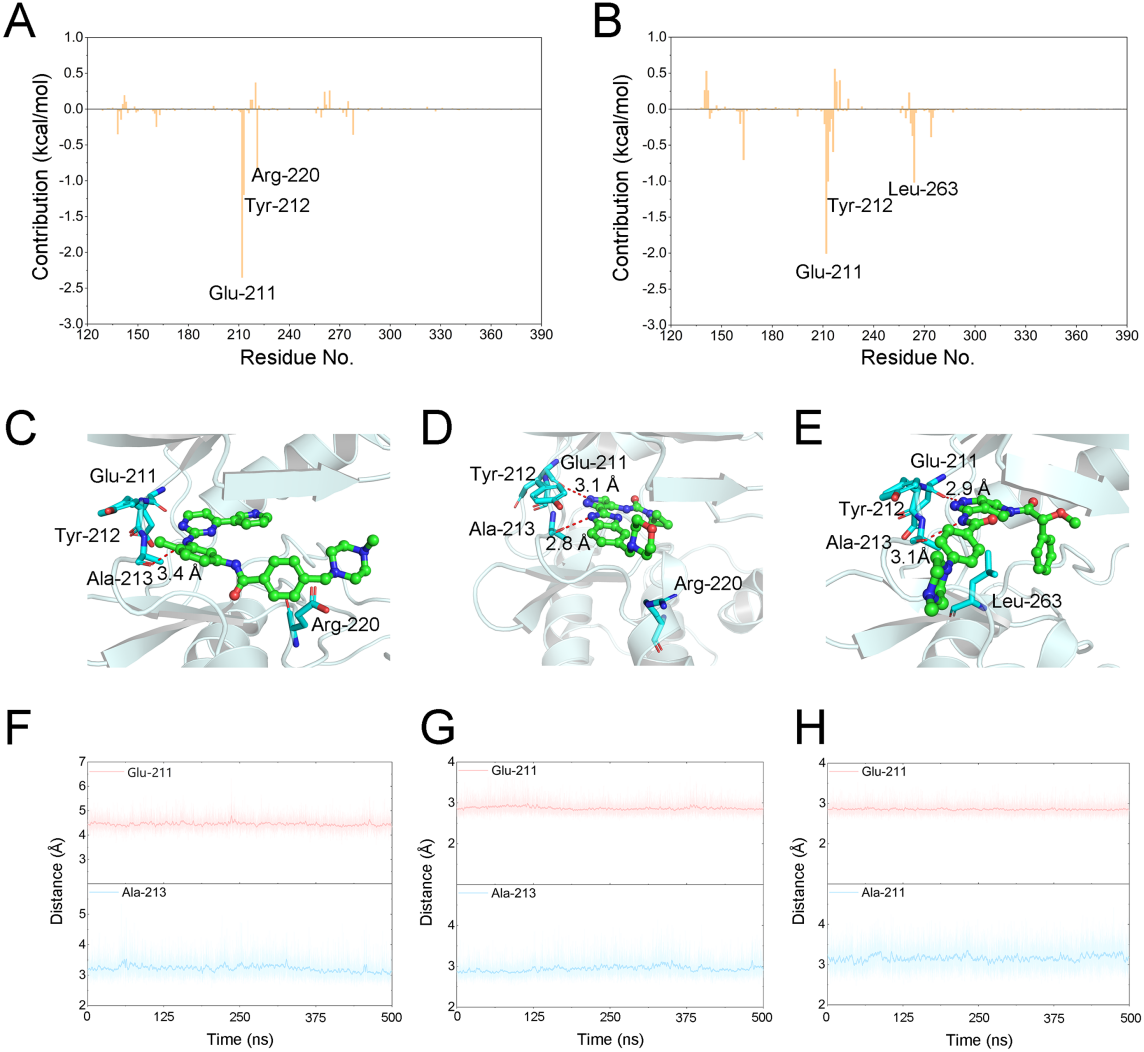
Energetic and structural analysis the differences between Gleevec and AT9283 or Danusertib. Differences of each residue contribution between Gleevec and AT9283 (A), Danusertib (B); Schematic view of the key residues of Gleevec (C), AT9283 (D), and Danusertib (E); The distance for the hydrogen bonds between Aurora A and Gleevec (nitrogen group of Gleevec to Glu-211, (F)), AT9283 (nitrogen groups of AT9283 to Glu-211 and Ala-213, (G)), and Danusertib (nitrogen groups of Danusertib to Glu-211 and Ala-213, (H)) as a function of time.

### Binding free energies calculations to determine the key residues

The binding free energy of Gleevec, AT9283 and Danusertib associated with Aurora A was calculated using Molecular Mechanics/Generalized Born Surface Area (MM/GBSA), which has been proved to be an important complement to the evaluation of protein-ligand interactions (Chen et al., 2018; Sun et al., 2014a; Sun et al., 2014b; Weng et al., 2019; Xu et al., 2013). In this study, although prediction of binding free energies based on multiple independent trajectories is more rigorous theoretically, the intramolecular energies (angle energy, bond energy *etc*.) cannot be well canceled by the multiple independent trajectories strategy. This might result in great uncertainty or noise of the calculated binding free energies (Δ*G*_binding_). Therefore, a single trajectory was performed following the procedure in previous studies (Sun et al., 2014b; Xue et al., 2014). As summarized in Table 1, the predicted Δ*G*_binding_ for Gleevec, AT9283 and Danusertib bound with Aurora A were -21.39 ± 3.11, −40.47 ± 2.90, and −38.99 ± 3.82 kcal/mol. These results were consistent with both of the reported experimental conclusions. Then, a detailed description of the binding of free energy to each residue in the receptor ligand recognition pattern was analyzed. Per-residue energetic differences between Aurora A bound with Gleevec and bound with AT9283 or Danusertib are plotted in Fig. 6. Positive values suggested that the interaction with Aurora A residue bound with Gleevec was stronger than the Aurora A residue bound with AT9283 or Danusertib. Conversely, negative values suggested that the interaction with Aurora A residue bound with Gleevec was weaker than the Aurora A residue bound with AT9283 or Danusertib. As shown in Fig. 6A, the residues of Glu-211, Tyr-212 and Arg-220 interacted more significantly with AT9283 in Aurora A than in Gleevec. Similarly, the differences between Aurora A/Gleevec and Aurora A/Danusertib were quite similar. When bound to Aurora A, the residues of Glu-211, Tyr-212 and Leu-263 interacted more significantly with AT9283 than with Gleevec (Fig. 6B). Notably, the residues of Glu-211 and Tyr-212 were located in the conserved structural elements of hinge region required for ligand binding (Figs. 6C–6E). Structural analysis of Aurora A/Gleevec revealed that at a distance of 3.4 Å, the nitrogen group of Gleevec formed a hydrogen bond with the backbone carbonyl of Ala-213. In contrast, binding of AT9283 or Danusertib with Aurora A revealed a different picture (Fig. 6C). The nitrogen groups of AT9283 and Danusertib formed important hydrogen bonds with the residues of Glu-211 and Ala-213. In particular, the Glu-211 was not formed for Gleevec (Figs. 6D–6E). To further analyze the stability of these hydrogen bonds, the distance evaluations of hydrogen bonds were monitored during the whole cMD simulation process and plotted (Figs. 6F–6H). As shown in Figs. 6F–6H, the distance of hydrogen bond for Gleevec to Ala-213 were relative stable within 3.5 Å during the 500 ns cMD simulations. Similarly, these for AT9283 and Danusertib were also quite stable during the simulations. However, the distances of hydrogen bond between Gleevec and Glu-213 were more than 4 Å during the whole cMD simulations. That is to say, Gleevec cannot form stable hydrogen bond with Glu-213 during the cMD simulation and cannot stabilize the hinge region. As a comparison, the distance of hydrogen bond for AT9283 and Danusertib to Glu-211 were quite stable within 3.5 Å during the whole cMD simulations. These observations suggested that the hydrogen-bond networks for AT9283 and Danusertib bound to Aurora A were more stable than the networks bound to Gleevec, which were congruent with the DCC analysis results that the hinge region were more dramatic (Fig. 3).

**Figure 7:**
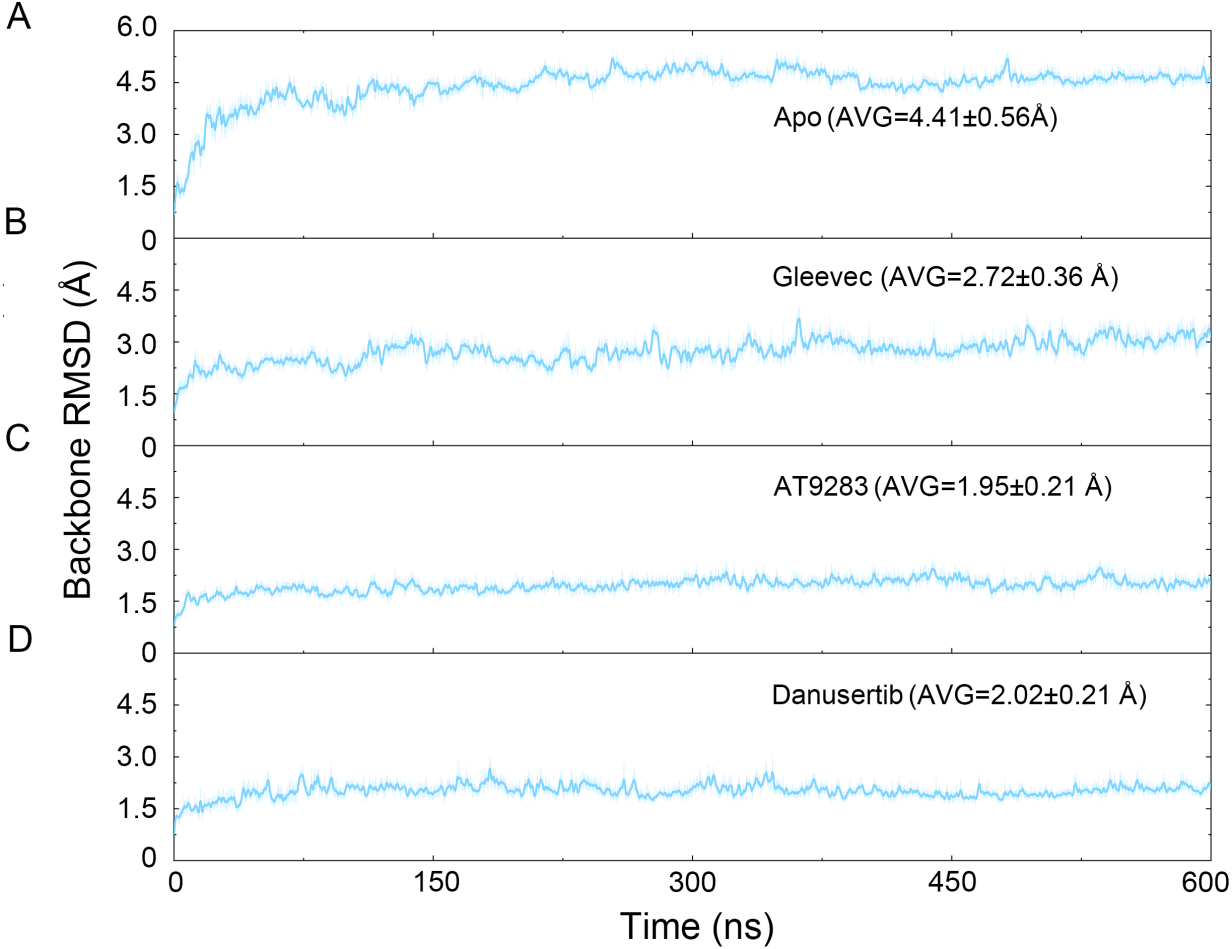
Time evolution of the RMSD values of backbone atoms of the Aurora A protein with (Gleevec, AT9283 and Danusertib) or without (apo) ligand in the simulated systems from GaMD simulations. (A) Apo Aurora A; (B) Aurora A/Gleevec; (C) Aurora A/AT9283; (D) Aurora A/Danusertib.

**Figure 8:**
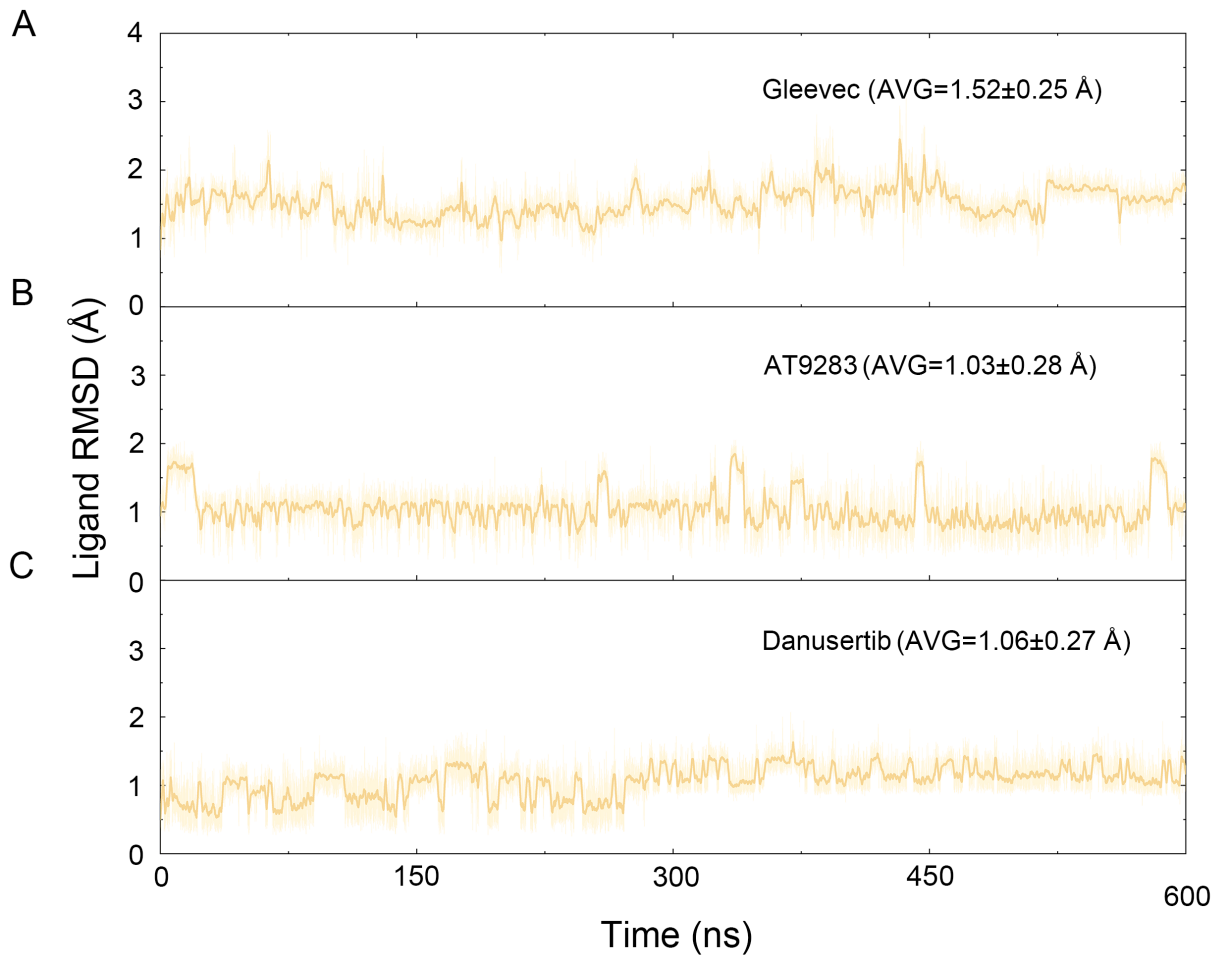
Time evolution of the RMSD values of heavy atoms of Gleevec, AT9283 and Danusertib bound with Aurora A in the simulated systems from GaMD simulations. (A) Aurora A/Gleevec; (B) Aurora A/AT9283; (C) Aurora A/Danusertib.

**Figure 9:**
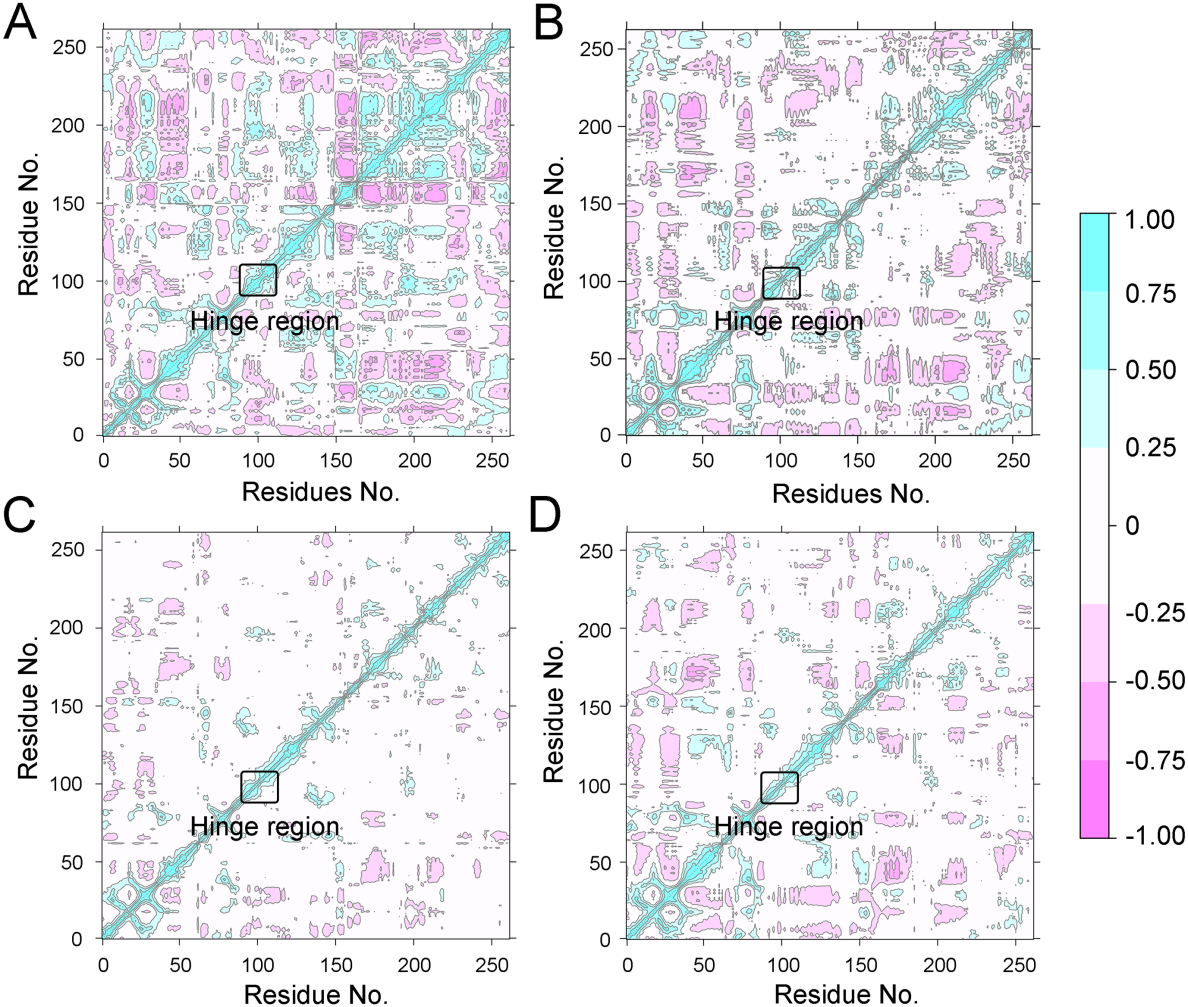
DCC analysis of fluctuations of residues from GaMD simulations. (A) Apo Aurora A; (B) Aurora A/Gleevec; (C) Aurora A/AT9283; (D) Aurora A/Danusertib.

### GaMD simulations supported the results from cMD simualtions

To better understand the free energy map (FEM) associated with the conformational changes of Aurora A coupled with different ligands, the conformation ensemble was sampled in more detail and the possible hidden energy barriers in the cMD simulation were detected. Subsequent to the GaMD simulations, the RMSDs of the protein backbone and the heavy atoms of the ligand were calculated. As shown in Fig. 7, the RMSDs of protein backbones in all the simulated systems reached equilibrium after ∼50–100 ns of GaMD simulations. The RMSDs of the heavy atoms of ligands in each simulated system remained dynamic (Fig. 8). Similar to the results from cMD simulations, the average RMSD values of protein backbone of Aurora A from GcMD simulations follow the order of Apo >Gleevec >Danusertib >AT9283, and the heavy atoms of ligands follow the order of Gleevec >AT9283 >Danusertib. In addition, DCC analysis indicated that the correlated and anti-correlated motions were stronger in the Apo Aurora A and Aurora A/Gleevec than those in Aurora A bound with AT9283 and Danusertib, which is consistent with the results from cMD simulations (Fig. 9). The above results indicated that Aurora A, when bound with non-selective inhibitors, induces a larger conformational fluctuation and greater variability between protein subunits. In contrast, when bound with selective inhibitors, Aurora A stabilizes the conformational ensembles.

**Figure 10:**
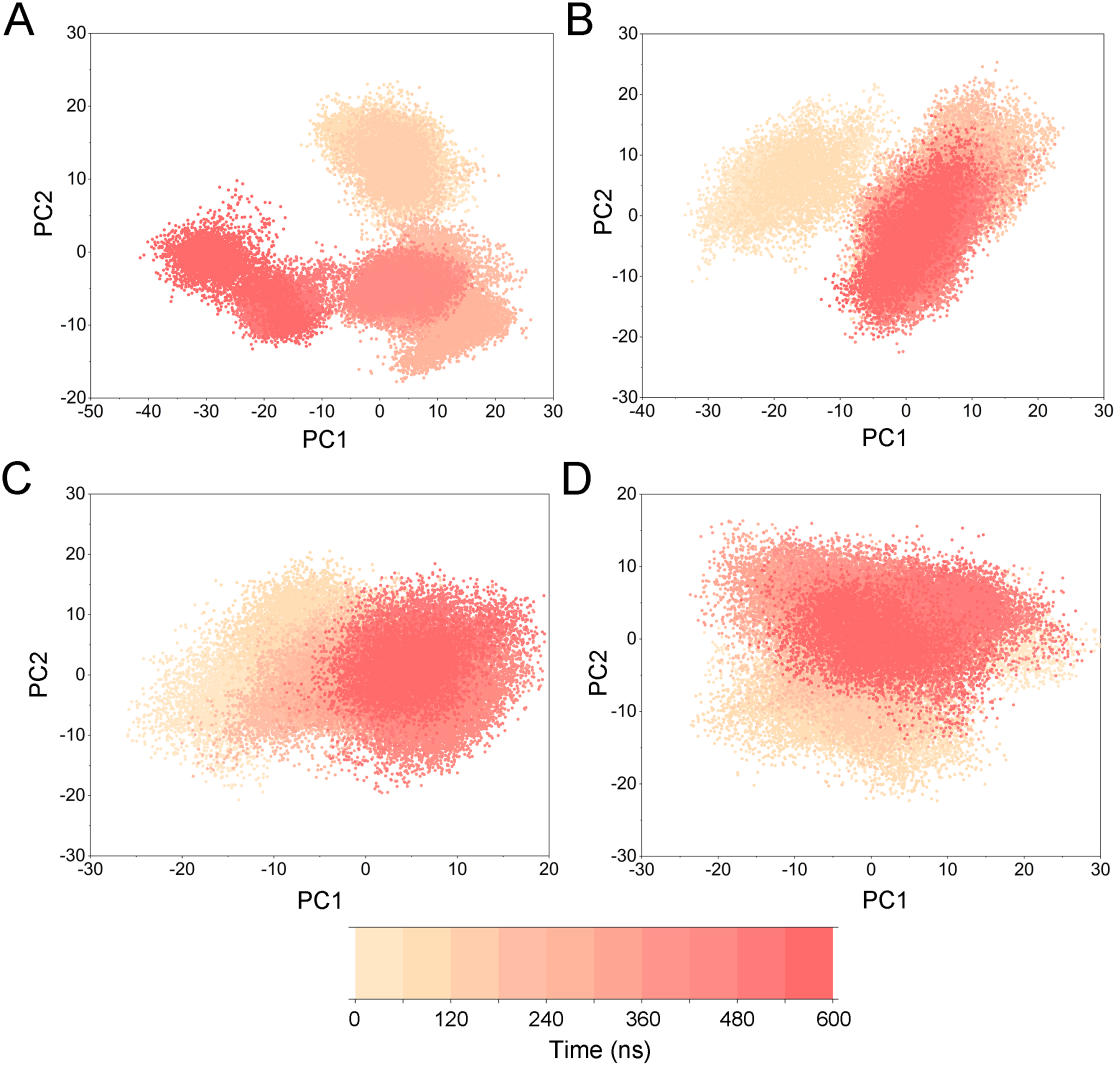
PCA scatter plot along the first two principal components from GaMD simulations. (A) Apo Aurora A; (B) Aurora A/Gleevec; (C) Aurora A/AT9283; (D) Aurora A/Danusertib.

**Figure 11:**
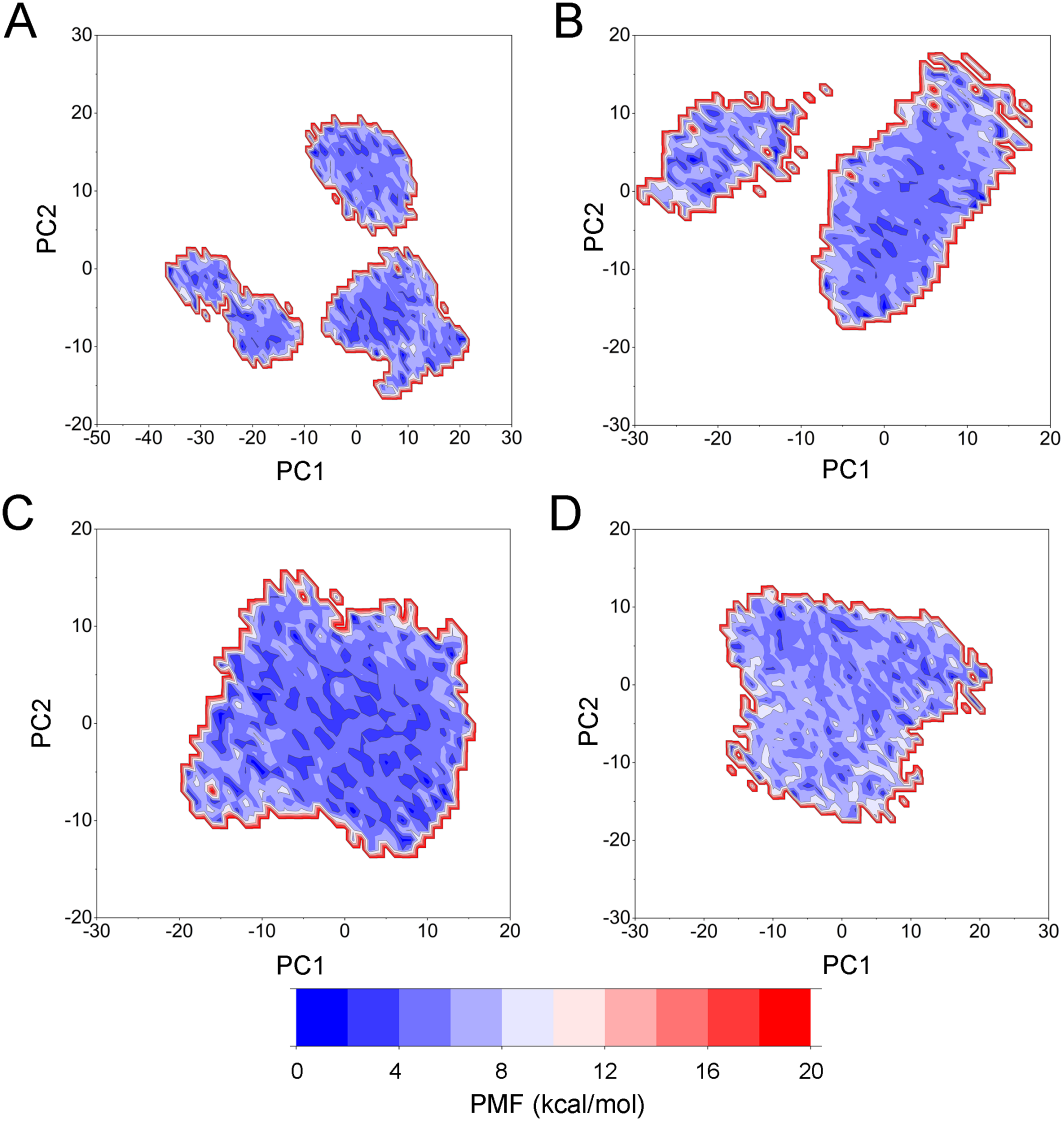
Free energy map along the first two principal components from GaMD simulations. (A) Apo Aurora A; (B) Aurora A/Gleevec; (C) Aurora A/AT9283; (D) Aurora A/Danusertib.

PCA was then performed to visualize the conformational changes. As shown in Fig. 10, the conformational distributions of Apo Aurora A and Aurora A/Gleevec were significantly different from Aurora A bound with AT9283 or Danusertib. Compared to the Apo Aurora A and Aurora A/Gleevec, the systems of Aurora A/AT9283 and Aurora A/Danusertib showed fewer structural clusters, suggesting that the selective inhibitors could stabilize the protein conformation. Thereafter, the FEM was performed to assess the relationship between the conformational changes and energy changes (Fig. 11). Theoretically, more energy wells (dark blue regions) indicated that the protein underwent larger conformational changes during MD simulations. As shown in Figs. 11A–11B, three and two major deep energy wells were observed for the Apo Aurora A and Aurora A/Gleevec. However, Aurora A bound with AT9283 or Danusertib were confined to a single deep energy well with high similarity, demonstrating the unstable nature of Aurora A bound with non-selective inhibitors during GaMD simulations (Figs. 11C–11D). These results are also supported by previous findings from cMD simulations, which showed that hinge region may directly regulate the conformational ensemble to ligand specifically binding.

### Dissociation pathways of Gleevec, AT9283 and Danusertib from Aurora A

**Figure 12:**
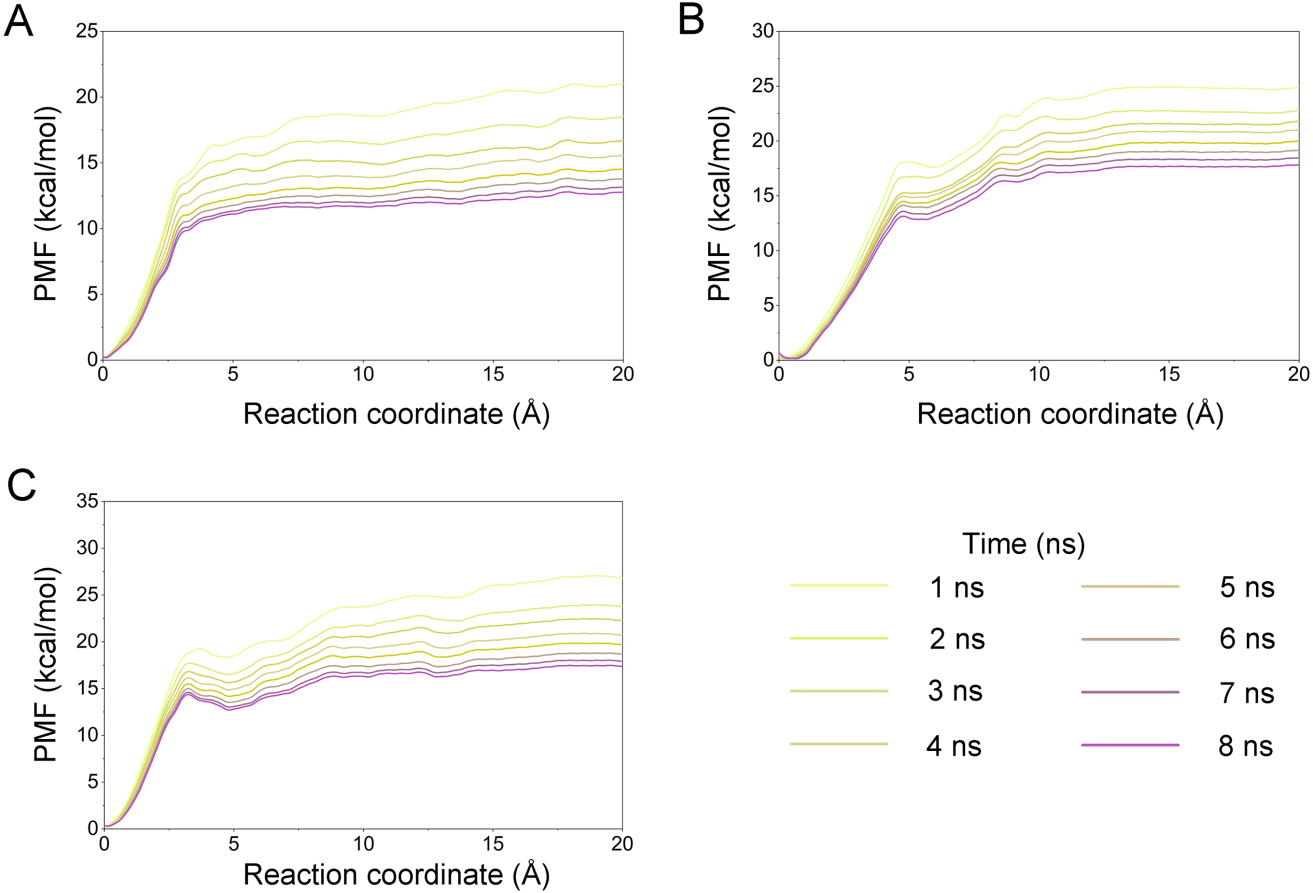
Convergence of the PMFs calculated by umbrella sampling simulations. (A) Aurora A/Gleevec; (B) Aurora A/AT9283; (C) Aurora A/Danusertib.

**Figure 13:**
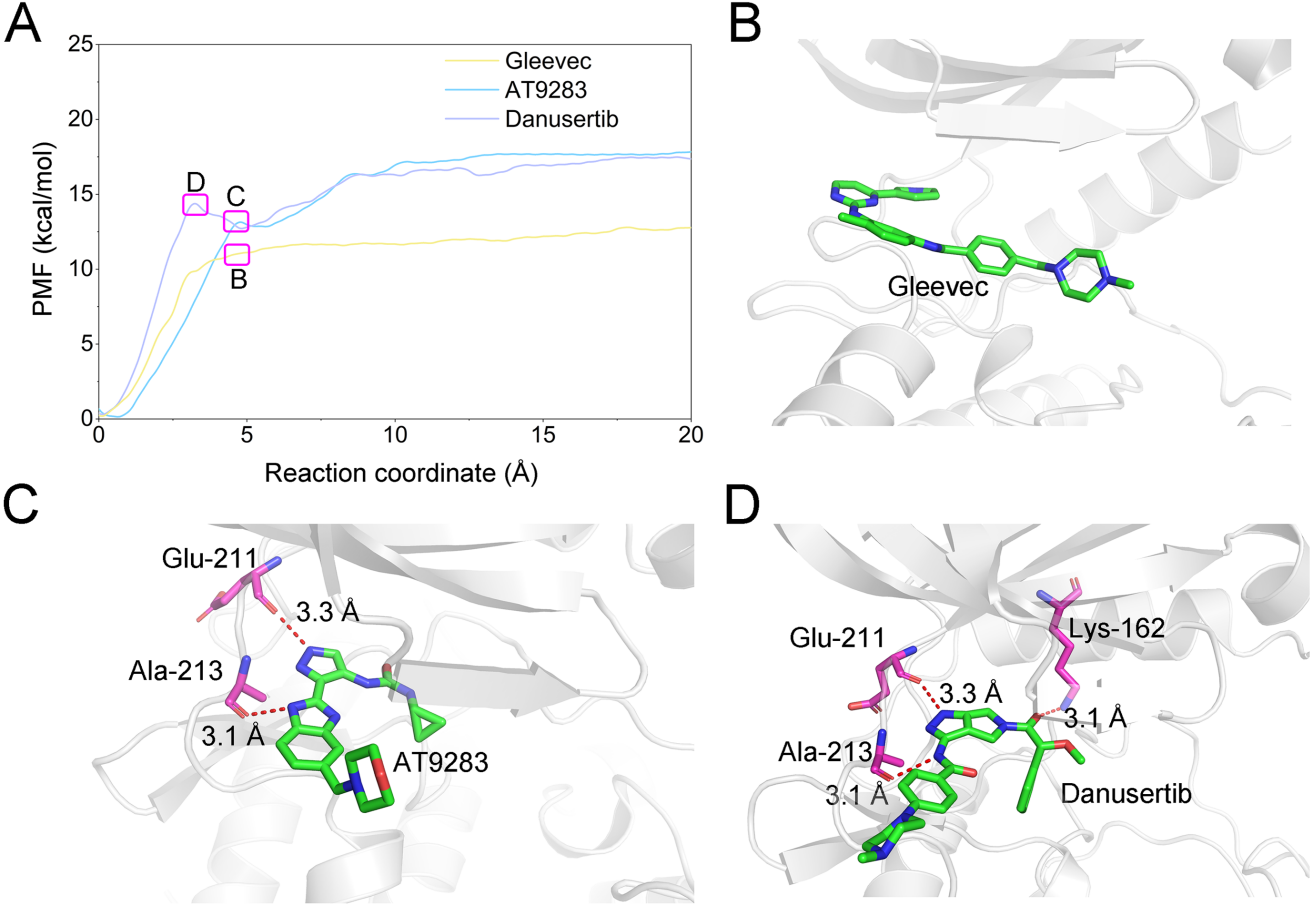
Comparison of the converged PMFs based on different complexes using the umbrella sampling simulations. (A) Comparison of the converged PMFs; Snapshots of Aurora A/Gleevec (B), Aurora A/AT9283 (C), and Aurora A/Danusertib (D).

A growing body of research has suggested that dissociation process of a ligand from its target can reveal the dynamic mechanisms of interactions, which is vital to the development of a novel selective ligand. Even though recent advances in computing power have allowed for the use of long-time-scale cMD simulations, simulation of the dissociation pathway of a ligand from its target remains difficult. Among the various enhanced sampling methods, the umbrella sampling simulation may be the most classic and commonly used method to explore the dissociation pathway of a specific ligand from the binding pocket from its target. In this study, the dissociation process of Gleevec, AT9283 and Danusertib from Aurora A was characterized by the umbrella sampling method. In order to guarantee the sampling convergence of the umbrella sampling simulations, each simulated window was submitted to 8 ns umbrella sampling simulations, and the convergence of potential of mean force (PMF) was checked every nanosecond. As illustrated in Fig. 12, eight curves were plotted for each simulated system, and the PMFs achieved satisfactory coincidence after 7 ns umbrella sampling simulations for each window (difference of PMFs, 0.5 kcal/mol). Thereafter, the binding affinities (PMF depth, Δ*W*_PMF_) were obtained by averaging the last 5 ns US simulations (21–25 ns). The Δ*W*_PMF_ of Aurora A/Gleevec, Aurora A/AT9283 and Aurora A/Danusertib were predicted by umbrella sampling simulations were, −12.73 ± 0.51, −17.83 ± 0.62, and −17.50 ± 0.60 kcal/mol, respectively. The Δ*W*_PMF_ can be congruent with the reported experimental data and correctly ranked. Thus, the PMF curves were utilized to clarify the different dissociation processes from the binding pocket of Gleevec, AT9283 and Danusertib toward Aurora A. The processes of the Gleevec dissociation from Aurora A were illustrated in Fig. 13 (yellow line). These results suggested that the PMF curves of AT9283 and Danusertib in Aurora A were obviously different from those of Gleevec, while the PMF values of AT9283 and Danusertib toward Aurora A were not significantly different. Initially, all the inhibitors were located at the binding sites of Aurora A (RC was 0 Å). When the inhibitors were dissociated from the binding site, the PMF values increased rapidly (RC was 0–10 Å). Thereafter, the PMFs along with the RC was on a steady increase toward equivalence (RC was 10–20 Å). During the rapid increase of the PMF values, the PMF curves of AT9283 and Danusertib showed an obvious peak and a valley. This phenomenon highlights the fact that the dissociations of AT9283 and Danusertib from Aurora A have an obvious energy barrier that needs to be overcome, and that no obvious energy barrier was observed for Gleevec. These observations indicated that the dissociation of AT9283 and Danusertib from Aurora A needs to overcome a higher free energy barrier than the Gleevec. Therefore, the AT9283 and Danusertib combined with Aurora A have a longer residence time. Further structural analysis showed that no hydrogen bond between Aurora A and Gleevec was observed when the RC were nearly 4 Å (Fig. 13B). In comparison, some important hydrogen bonds were observed for AT9283 and Danusertib at the peaks of the RCs. In addition, most of these hydrogen bonds were triggered by Glu-211, and Ala-213. These results are in agreement with previous findings based on the per-residue free energy decomposition and structural analysis, where the hydrogen-bond networks for Gleevec bound to Aurora A were not as stable as those for AT9283 and Danusertib, which means that AT9283 and Danusertib, but not Gleevec, could selectively bound to Aurora A.

## Conclusions

This study combined cMD simulations, GaMD simulations, umbrella sampling simulations and various analytical methods to illustrate the molecular principles of the binding selectivity of three inhibitors towards Aurora A, namely, Gleevec, AT9283 and Danusertib. The results from the cMD simulations provide compelling evidence that preferential binding of AT9283 and Danusertib to Aurora A was regulated by the conformational changes of kinase hinge region, which decreased the hydrogen bond interactions to key residue Glu-211. The GaMD simulations further supported these findings, indicating that Gleevec bound to Aurora A decreased the stability of Aurora A. In addition, the umbrella sampling simulations further confirmed the predictions of selectivity, as shown by the structurally and energy analysis from the cMD and GaMD simulations. Taken together, the results in this study offer theoretical insights into the selective mechanisms of inhibitors bound to Aurora A, which may prove conducive to the development of novel selective inhibitors targeting Aurora A.

##  Supplemental Information

10.7717/peerj.7832/supp-1 Figure S1 Reaction coordinates (RCs) of umbrella sampling simulations (A) Gleevec, (B) AT9283; (C) Danusertib.

10.7717/peerj.7832/supp-2 Figure S2 Statistics of the frequency of PCA scatter plots from cMD simulations (A) Apo Aurora A; (B) Aurora A/Gleevec; (C) Aurora A/AT9283; (D) Aurora A/Danusertib.

10.7717/peerj.7832/supp-3 Figure S3 Alignment of the highest frequency structures (magenta) and initial structures (gray) provided by PCA analysis from cMD simulations (A) Apo Aurora A; (B) Aurora A/Gleevec; (C) Aurora A/AT9283; (D) Aurora A/Danusertib.

10.7717/peerj.7832/supp-4 Data S1 Raw data The raw data are corresponding to figure order. pse and pdb format files can be opened in PyMol 1.8. cdx files can be opened in Chemdraw 16.0. csv files can be opened in Excel.

## Additional Information and Declarations

### Data Availability

The following information was supplied regarding data availability: The raw data are available in the Supplemental Files.
