# Supplementary figures and images for: Exploration of the selective binding mechanism of protein kinase Aurora A selectivity via a comprehensive molecular modeling study
Source: PeerJ. 2019 Oct 22;7:e7832. doi: 10.7717/peerj.7832 (PMC6814069; doi:10.7717/peerj.7832)

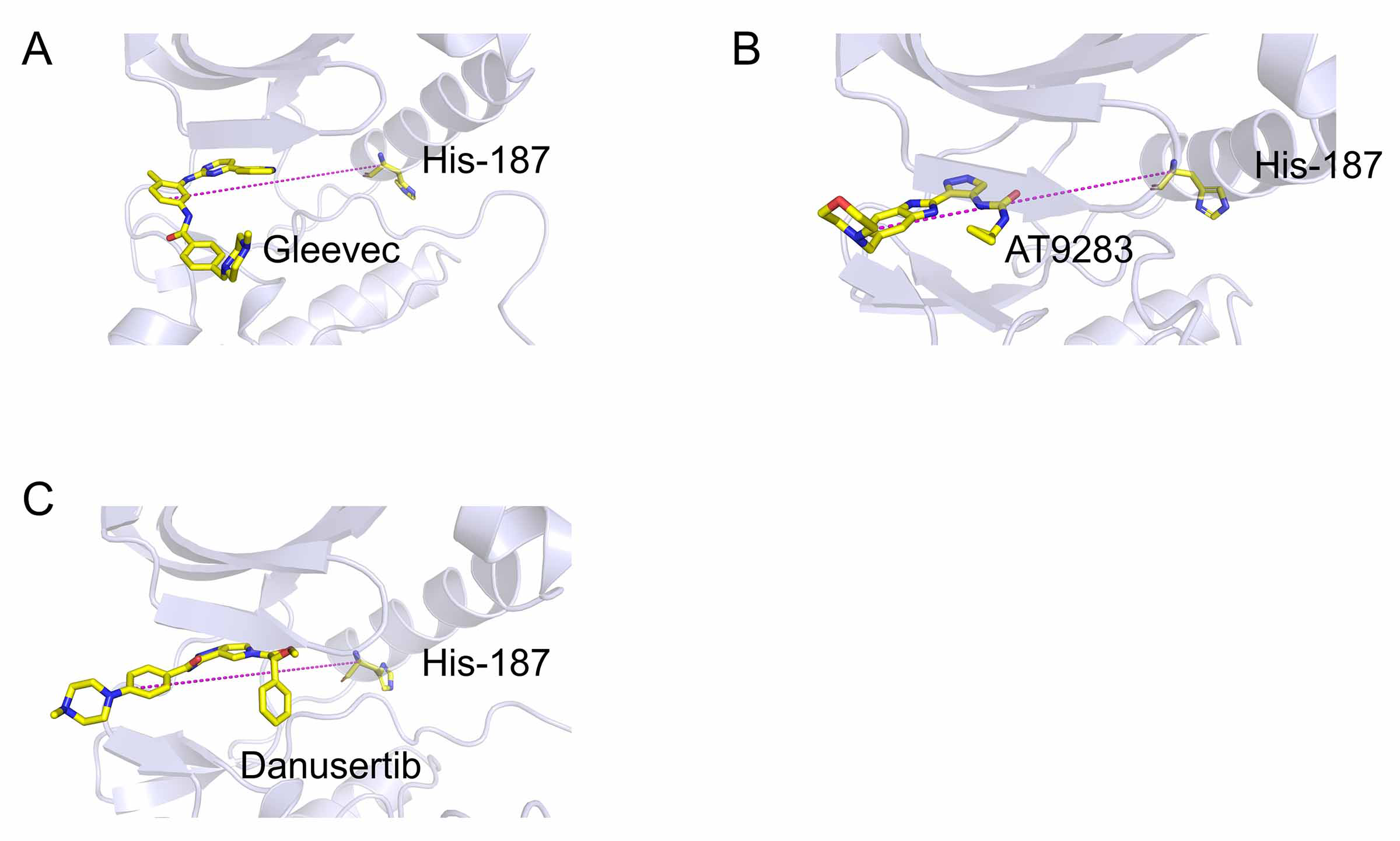

Supplement: Figure S1 — (A) Gleevec, (B) AT9283; (C) Danusertib. [file peerj-07-7832-s001.png]

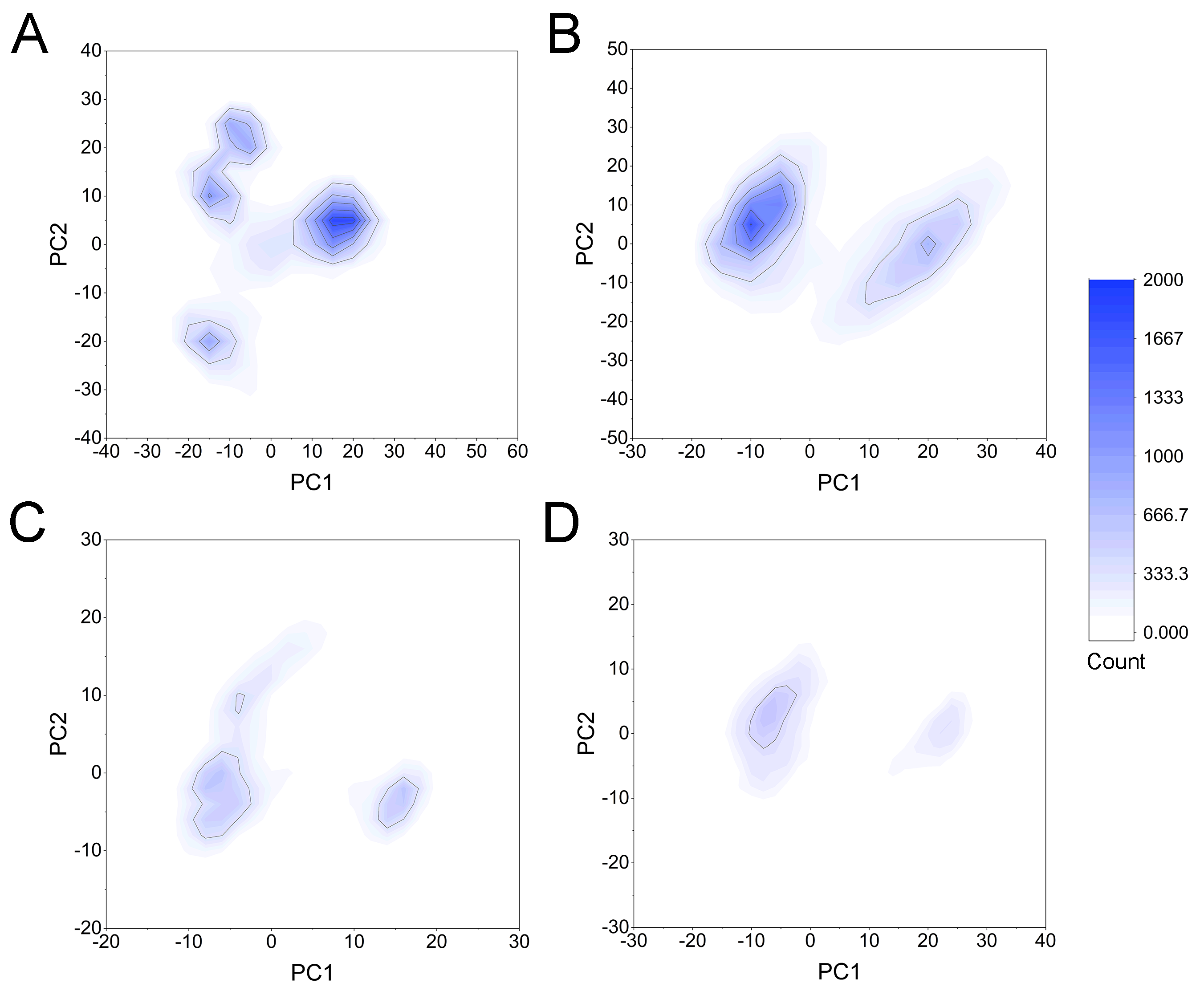

Supplement: Figure S2 — (A) Apo Aurora A; (B) Aurora A/Gleevec; (C) Aurora A/AT9283; (D) Aurora A/Danusertib. [file peerj-07-7832-s002.png]

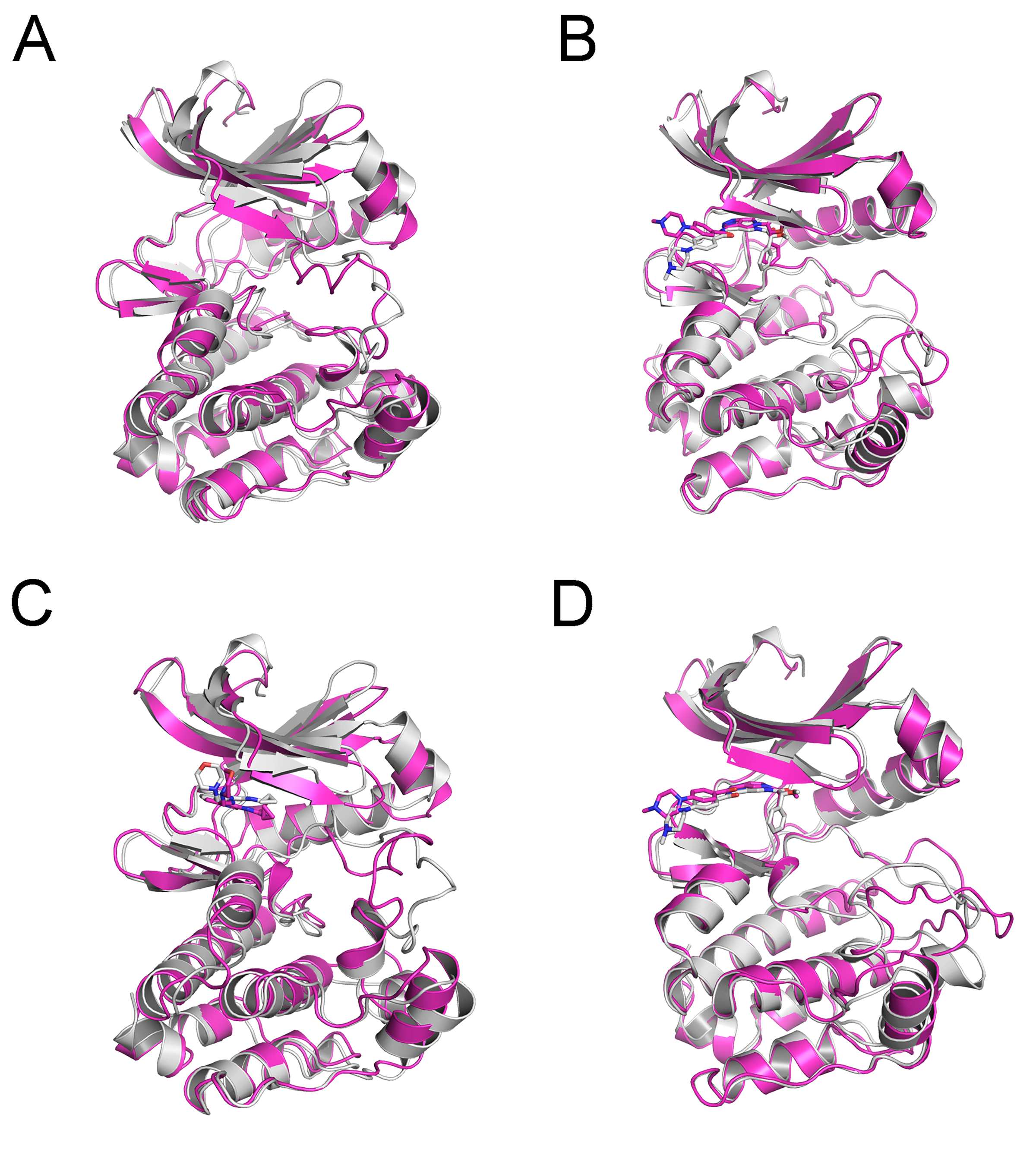

Supplement: Figure S3 — (A) Apo Aurora A; (B) Aurora A/Gleevec; (C) Aurora A/AT9283; (D) Aurora A/Danusertib. [file peerj-07-7832-s003.png]
